# Assessment of Polymyxin B with Sodium Deoxycholate Sulfate Micelles in a Rat Model to Combat Polymyxin Nephrotoxicity

**DOI:** 10.3390/antibiotics14111062

**Published:** 2025-10-23

**Authors:** Muhammad Ali Khumaini Mudhar Bintang, Jongdee Nopparat, Teerapol Srichana

**Affiliations:** 1 Drug Delivery System Excellence Center, Department of Pharmaceutical Technology, Faculty of Pharmaceutical Sciences, Prince of Songkla University, Hat Yai 90112, Thailand; khumaini@utnd.ac.id; 2 Division of Health and Applied Sciences, Faculty of Science, Prince of Songkla University, Hat Yai 90112, Thailand; jongdee.n@psu.ac.th

**Keywords:** Polymyxin B, sodium deoxycholate sulfate, nephrotoxicity, histopathology, biodistribution

## Abstract

**Background/Objectives:** Polymyxin B (PMB) was incorporated into a sodium deoxycholate sulfate (SDCS) micelle formulation to mitigate polymyxin-induced nephrotoxicity. This study examined the effect of the formulation on nephrotoxicity and biodistribution in a rat model. **Methods**: Four groups of rats were subcutaneously administered one of the following: normal saline, SDCS, PMB, or a PMB-SDCS formulation. After treatment, the weight changes were recorded, and the rats were euthanized to collect blood for serum biochemistry measurements. The histopathological damage to organs was examined. Two additional groups of rats received the same dose of PMB and the PMB-SDCS formulation subcutaneously; however, their serum PMB was measured at predetermined time points, and the PMB concentrations in the organs were measured. Molecular docking for PMB and formulation with human serum albumin was also performed. **Results**: The PMB-SDCS formulations showed improvement in serum biomarker measurements. Several abnormalities were observed in the kidney, liver, lung, and spleen tissues following PMB treatment, which indicated evidence of toxicity. The docking showed SDCS reduces PMB binding affinity on HSA. The PMB-SDCS formulations were associated with less acute toxicity and less nephrotoxic damage compared with the PMB group. The results were supported by less PMB accumulation in the kidneys in the formulation group. **Conclusions**: The study indicates that SDCS has the potential to mitigate PMB-induced nephrotoxicity in rat models, suggesting a promising strategy for safer use that warrants further investigation.

## 1. Introduction

According to the latest Global Research on Antimicrobial Resistance (GRAM) report, bacterial antimicrobial resistance was directly responsible for approximately 1.14 million deaths in 2021 and associated with 4.95 million deaths globally in 2019, highlighting the urgent need for effective therapies [1]. The prevalence of infections caused particularly by Gram-negative bacteria, such as multidrug-resistant *Acinetobacter baumannii*, *Klebsiella pneumoniae*, and *Pseudomonas aeruginosa*, has led to the re-emergence of polymyxins in clinical practice because they have proven to be effective against resistant Gram-negative strains [2,3]. Polymyxin B (PMB), which is a polypeptide antibiotic, predominantly targets the lipid A component of lipopolysaccharides (LPS) within the outer membrane of Gram-negative bacteria, thereby compromising the structural integrity of the membrane. Its self-uptake mechanism is critically reliant on its amphipathic properties that facilitate increased membrane permeability and result in leakage of intracellular contents [4].

Nephrotoxicity remains a significant challenge for PMB. The prevalence of nephrotoxicity ranges from 10% to 60% despite the effectiveness of PMB in treating multidrug-resistant Gram-negative bacterial infections [5,6]. The amphipathic properties of polymyxins are also responsible for their nephrotoxicity where the cationic diaminobutyric acid (Dab) residues of PMB interact with anionic phospholipids (e.g., phosphatidylinositol, cardiolipin) on the membranes of renal tubular cells. This electrostatic interaction, combined with the insertion of the lipophilic tail into the lipid bilayer, disrupts membrane integrity, increases permeability, and leads to cell swelling, necrosis, and ultimately, nephrotoxicity [7,8]. In contrast, PMB nonapeptide (PMB without the fatty acyl tail) was found to uniformly distribute in the kidney [9]. The selective uptake with high tubular reabsorption was shown in low urinary recovery (<5%) from animal models [7,10]. Studies suggest that the toxicity to renal tubular cells is indicated by rising creatinine (Cr) levels in the blood. The proposed mechanisms include oxidative stress, apoptosis, cell cycle arrest, and autophagy. Oxidative stress disrupts redox balance and causes lipid peroxidation, protein damage, and mitochondrial dysfunction, while apoptosis leads to tubular cell loss via caspase activation and cytochrome c release [11,12].

There is a high demand for new antimicrobial agents to combat resistant bacteria; however, the complex processes, regulatory challenges, and financial risks have made research into nanotechnology-based antimicrobial drugs more preferable [13,14,15]. The development of a suitable delivery system would aim to optimize the pharmacokinetics and pharmacodynamics of PMB to reduce systemic exposure and deliver the drug more precisely to infection sites to mitigate the toxicity of PMB and enhance antimicrobial performance [16]. The chemical structure and properties of polymyxins are important factors to consider in designing a delivery system. The five primary aliphatic amino groups in polymyxins facilitate their efficient binding with anionic molecules and polyanions. Additionally, the combination of a positively charged structure, a lipophilic tail, and hydrophobic amino acids in the peptide chain enables the use of amphiphilic and hydrophobic low-molecular-weight compounds and polymers as carriers in drug delivery systems [17,18,19].

Micelle-based delivery systems have shown significant promise in enhancing the therapeutic efficacy of peptide antibiotics. These nanosized carriers, which are formed by the self-assembly of amphiphilic molecules, can encapsulate antimicrobial peptides to improve solubility, stability, and bioavailability [20]. Sodium deoxycholate sulfate (SDCS) is an amphiphilic carrier that is synthesized from deoxycholic acid [21]. SDCS is particularly suitable for delivering PMB due to its unique structural properties. As an anionic surfactant, it can form stable micellar complexes with the cationic PMB molecule via electrostatic and hydrophobic interactions. Its safety profile has been demonstrated in previous in vitro and in vivo studies, which showed that SDCS formulations of nephrotoxic drugs like amphotericin B and colistin significantly reduced cytotoxicity and organ damage without compromising antimicrobial efficacy [22,23,24,25]. For instance, PMB-SDCS formulations demonstrated significantly reduced cytotoxicity in human proximal tubule (HK-2) cells while retaining potent antimicrobial activity against carbapenem-resistant *Acinetobacter baumannii* and *Pseudomonas aeruginosa* [23,26]. Rats that were administered amphotericin B and SDCS formulations via intratracheal instillation showed no evidence of toxicity, especially in lung and kidney tissue, with significantly lower drug accumulation in the kidney compared with a commercial formulation [25]. A prior study showed that PMB-SDCS formulations facilitated PMB release for effective penetration into lipid membranes or micelles. The complex LPS micelles or membranes were disrupted while neutralizing LPSs through hydrogen bonding and electrostatic interactions between PMB amine residues and the phosphate and glucosamine groups of LPSs, as well as salt bridge formation with SDCS sulfonate groups [27]. The PMB-SDCS complex is formed primarily through electrostatic interactions between the cationic Dab residues of PMB and the anionic sulfate groups of SDCS, supplemented by hydrophobic interactions between the fatty acyl tail of PMB and the steroid backbone of SDCS. This results in the encapsulation of PMB within the SDCS micelles, enhancing its aqueous stability and altering its interaction with biological membranes [27,28].

The objective of the present study was to investigate PMB-SDCS formulation treatment effects in a rat model. The treatments caused histological alterations in the kidney and several other organs. Biochemical markers that indicated the treatments’ nephrotoxicity were measured. PMB concentrations in the serum and tissue were also analyzed to further investigate the formulation’s effect in vivo, while using molecular docking to analyze the human serum albumin (HSA) interaction. The binding affinity of PMB to HSA is a key pharmacokinetic parameter, as extensive binding can reduce the free, pharmacologically active drug concentration, potentially necessitating higher doses that increase the risk of nephrotoxicity. Investigating this interaction helps elucidate the formulation’s mechanism for increasing/decreasing serum drug availability.

## 2. Results

### 2.1. PMB Formulation Preparation and Properties

The PMB formulation was prepared by incorporating PMB in SDCS micelles and freeze-drying the solution, which yielded a white, very light, and free-flowing powder. The dry powder was reconstituted before use to produce a clear solution with a pH of 7.4. Table 1 shows the characteristics of the PMB-SDCS formulation in a 1:2 molar ratio that was chosen from a previous study [23,26]. The hydrodynamic properties of the reconstituted PMB formulation were measured by dynamic light scattering, where the particle size was 193 ± 27 nm with a zeta potential of −5.84 ± 1.32 mV. The PMB concentration in the formulation was measured using LC-MS, with the PMB-SDCS micelle formulation having a 57.27% *w*/*w* drug content. This result was used to calculate the dose administered in vivo. The osmolarity of the reconstituted formulation in normal saline was found to be 311 ± 2 mOsm/Kg.

### 2.2. Effect of the Treatment on Rat Body Weight Gain

**Figure 1:**
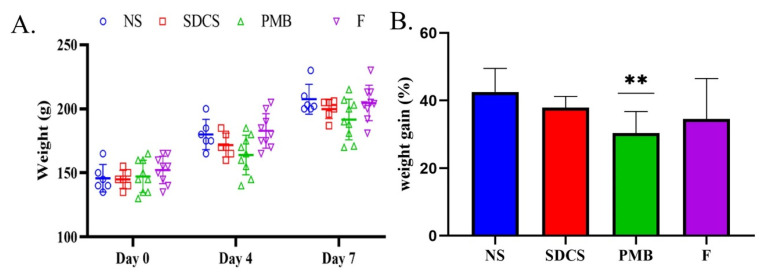
Effect of the PMB-SDCS formulation (F) on (**A**) the weight change measured at days 0, 4, and 7, and (**B**) the percentage of body weight gain after 7 days of treatment. ** *p* < 0.01 (mean ± SD, *n* = 9).

After seven days of treatment with 6 mg/kg of the PMB and PMB formulation, the weight progression of the rats in each group was measured and recorded. All 36 rats successfully acclimated and reached the target body weight of 110 g within the 7-day period. No animals were excluded from the study. The results in Figure 1A show the weight changes measured on day 0, day 4, and day 7 of administration. The body weight of the PMB-treated group was significantly lower on day 4 (163.89 ± 15.37 g vs. 180.0 ± 11.83 g in the normal saline group, *p* < 0.05) and day 7 (191.67 ± 15.83 g vs. 207.5 ± 11.62 g in the normal saline group, *p* < 0.01) compared to the other treatment groups. Statistical analysis confirmed that the PMB group exhibited a significant reduction in body weight gain compared with both the control and PMB-SDCS groups (*p* < 0.05). No significant difference was observed between the PMB-SDCS and control groups (*p* > 0.05).

### 2.3. Kidney Function of the Treated Rats

**Figure 2:**
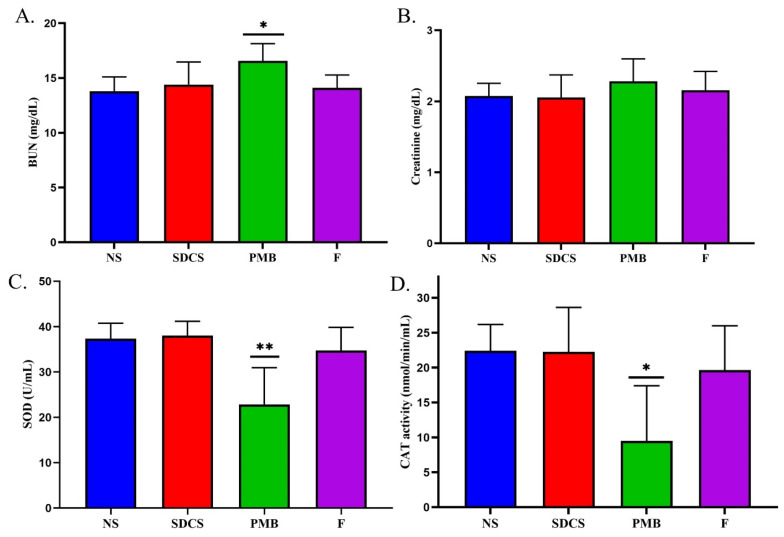
Effect of the PMB and PMB-SDCS formulation (F) on rat kidney function and serum oxidative stress: (**A**) blood urea nitrogen (BUN); (**B**) creatinine; (**C**) superoxide dismutase (SOD); and (**D**) catalase (CAT). Data are presented as mean ± SD (*n* = 5). The asterisk(s) represent statistical differences from the control group. * *p* < 0.05 and ** *p* < 0.01.

After seven days of treatment, the serum levels of blood urea nitrogen (BUN) and Cr were assessed in the rats to investigate their kidney function. The results in Figure 2A show a significant increase in the BUN levels after the PMB treatments (16.56 ± 1.59 mg/dL) compared to the normal saline group (13.8 ± 1.3 mg/dL) (*p* < 0.05) with no significant difference between the PMB formulation group (14.11 ± 1.17 mg/dL) and the SDCS group (14.4 ± 2.07 mg/dL). The Cr levels compared to the normal saline group (2.08 ± 1.08 mg/dL) was not significantly different between the formulation group (2.15 ± 0.27 mg/dL) and the SDCS group (2.06 ± 0.32 mg/dL). The PMB group had slightly elevated Cr levels (2.28 ± 0.31 mg/dL) (Figure 2B).

### 2.4. Oxidative Stress Biomarkers of the Treated Rats

The serum levels of superoxide dismutase (SOD) and Catalase (CAT) in rats were analyzed to evaluate and compare the oxidative stress induced by the treatments. The normal saline group exhibited a serum SOD level of 37.36 ± 3.4 U/mL. No significant reductions were detected in the serum SOD levels of the SDCS (38 ± 3.17 U/mL) and formulation (34.73 ± 5.17 U/mL) groups. However, the PMB-treated group demonstrated a significantly lower serum SOD level (22.87 ± 8.1 U/mL) (*p* < 0.01) compared to the control group (Figure 2C). The serum CAT levels in the treated rats followed a trend similar to that observed for the SOD levels (Figure 2D). In the formulation (19.65 ± 6.35 nmol/min/mL) and SDCS (22.25 ± 6.38 nmol/min/mL) groups, no significant differences in serum CAT levels were observed compared to the control group (22.4 ± 3.79 nmol/min/mL). However, the PMB-treated rats exhibited a significantly lower serum CAT level (9.5 ± 7.91 nmol/min/mL) (*p* < 0.01), which indicated a pronounced effect of the treatments on oxidative stress markers.

### 2.5. Histopathological Examination

**Figure 3:**
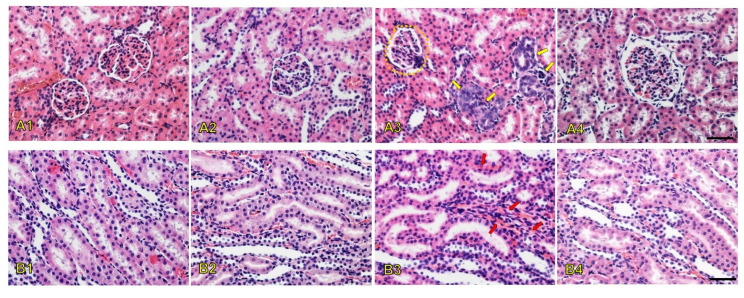
Kidney histopathological alterations in the glomerulus in the renal cortex (**A1**–**A4**) and renal tubules in the renal medulla (**B1**–**B4**) among the treatment groups. (**A1**,**A2**,**B1**,**B2)**: normal kidney tissue histopathology from the normal saline and SDCS groups, respectively. (**A3**,**B3**): The PMB group shows deformation of the glomeruli (yellow circle), inflammatory cell infiltrations (yellow arrows), and dilation and congestion of the vasa recta (red arrows). (**A4**,**B4**): The PMB formulation group shows mild alternations without evidence of renal glomerular injury. Magnification: ×20. Scale bar: 100 µm.

An H&E assay was performed on the collected tissues of all animals to evaluate PMB-induced nephrotoxicity and organ damage. The kidney histopathological results from the H&E assay of the renal cortex and medulla in the treated animals are shown in Figure 3. The histological data revealed that normal glomeruli, proximal tubular cells, and vessels were present in the kidneys of the normal saline group (Figure 3A1,B1). In addition, the histological examination of the kidney showed that the morphology of the SDCS-treated rats was also normal (Figure 3A2,B2). Deformation of the glomeruli and congestion of the renal blood vessels were prominently observed in the PMB-treated animals (Figure 3A3,B3). In contrast, the PMB formulation group exhibited only mild alterations and showed no significant signs of renal glomerular injury (Figure 3A4,B4).

**Figure 4:**
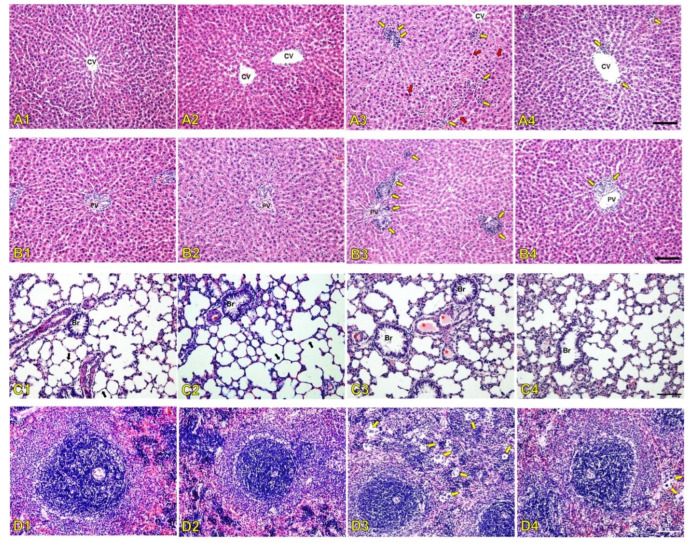
Organ tissue histopathology alterations from the normal saline (NS) (**A1**–**D1**), SDCS (**A2**–**D2**), PMB (**A3**–**D3**), and PMB formulation (**A4**–**D4**) groups. The liver tissue sections with H&E staining (**A**,**B**) show normal liver alteration in the NS and SDCS groups. Infiltration of monocytes (yellow arrows) with loss of hepatocyte architecture and necrotic foci (red arrows) around the blood vessels are observed in the PMB group. The histological changes were less severe with a few monocyte invasions in the PMB formulation-treated animals (yellow arrow). The lung tissue (**C**) shows normal lung tissue histology from the NS and SDCS groups. Lung parenchyma in the PMB rats demonstrated an increased number of small pulmonary venules containing fibrin thrombi (red asterisks) that indicate endothelial damage. A few thrombi can be observed in the lung in the PMB formulation-treated animals (red asterisks). The spleen tissue (**D**) shows normal white and red pulp from NS and SDCS groups. A large number of multinucleated giant cells (yellow arrows) are present in the PMB-treated animals. Fewer numbers of multinucleated giant cells are observed in the spleen in PMB formulation-treated animals (yellow arrows). Magnification: ×20. Scale bar: 100 µm. CV central vein, PV portal vein.

Liver histopathological alterations in the central vein area and portal triad area of the treated rats are shown in Figure 4A and Figure 4B, respectively. Histopathological findings showed normally organized hepatocytes that formed radiating hepatic cords around the central vein with normal hepatic tissues, including blood sinusoids and portal structures that were observed in tissue sections from the normal saline group (Figure 4A1,B1) and SDCS group (Figure 4A2,B2). The liver histology of the PMB-treated animals revealed significant monocyte infiltration accompanied by loss of hepatocyte architecture and the presence of necrotic foci around the blood vessels (Figure 4A3,B3). In contrast, the histological changes in the PMB formulation group were less severe, showing only minimal monocyte infiltration (Figure 4A4,B4).

The histology analysis of the lung tissue (Figure 4C) showed normal lung tissue characterized by a thin alveolar-capillary membrane separating red blood cells from the alveolar space, and including endothelial cells, basal membrane, and epithelial cells in the normal saline and SDCS groups (Figure 4C1,C2). The lung parenchyma of the PMB-treated rats showed endothelial damage that was evidenced by an increased number of small pulmonary venules containing fibrin thrombi that indicated vascular injury (Figure 4C3). A few thrombi were observed in the lung in the PMB formulation group (Figure 4C4).

Histopathological alterations in the spleen tissue of the treated animals were also examined (Figure 4D). The spleens of the normal saline and SDCS groups displayed normal white and red pulp that was characterized by predominantly non-activated follicles and unremarkable red pulp (Figure 4D1,D2). In contrast, the PMB group showed numerous multinucleated giant cells in the spleen tissue (Figure 4D3), while fewer multinucleated giant cells were observed in the spleen tissue of the PMB formulation-treated animals (Figure 4D4).

### 2.6. SDCS Affected the Biodistribution and Serum Concentration of PMB

**Figure 5:**
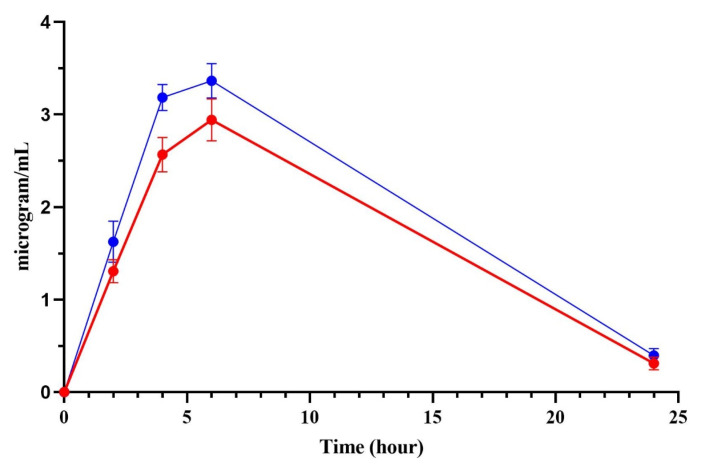
PMB serum concentrations at different time points after PMB (red) and PMB-SDCS formulation (blue) subcutaneous administration (6 mg/kg) (mean ± SD, *n* = 4).

**Figure 6:**
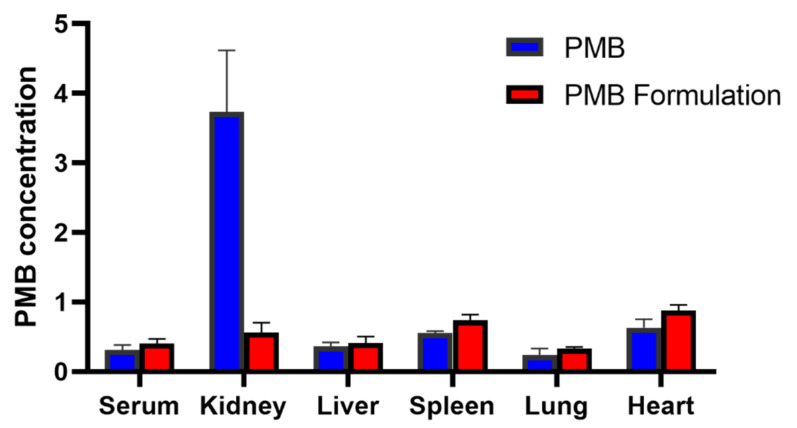
PMB concentrations in serum (µg/mL) and tissues (µg/g) after 24 h of subcutaneous administration of PMB and PMB-SDCS formulation (6 mg/kg/day) (mean ± SD, *n* = 4).

Following the subcutaneous administration of 6 mg/kg of either PMB or PMB-SDCS formulation in rats, the concentrations of PMB in the organs and serum were analyzed. Figure 5 shows the serum concentrations of PMB at different time points. There are noticeable differences in the amounts of free PMB between the rats administered with pure PMB compared to the PMB-SDCS formulation. The serum concentrations reported represent the free (unbound) PMB fraction determined after protein precipitation and filtration, which is the pharmacologically active component. The differences are apparent at the second and fourth hours where PMB concentrations were 2.57 ± 0.19 and 2.94 ± 0.23 µg/mL for rats administered with pure PMB, while the PMB-SDCS group showed 3.18 ± 0.14 and 3.36 ± 0.18 µg/mL, respectively. This high level of free PMB in the serum from the PMB-SDCS-treated rats directly affected the PMB disposition in the organs of the rats. Figure 6 shows the biodistribution of PMB in several organs and serum after 24 h. Significant concentrations of PMB (3.73 ± 0.89 µg/g) were found in the kidneys of the PMB-treated rats, while the PMB-SDCS-treated rats had significantly lower concentrations of PMB (0.56 ± 0.14 µg/g).

### 2.7. SDCS Effect on PMB Binding in HSA

**Figure 7:**
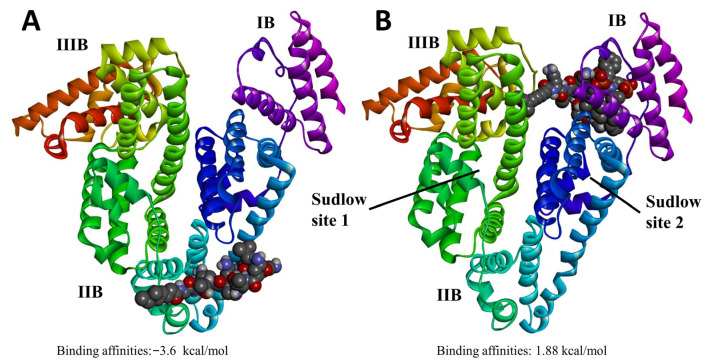
The binding conformation of (**A**) PMB docked on HSA and (**B**) PMB-SDCS docked on HSA. PMB binds at a surface pocket near subdomain IIB (non-Sudlow site). The conformation and binding affinities were the highest-ranked conformation.

The molecular docking was performed to simulate the interaction of PMB and HSA affected by SDCS. The molecular docking showed that PMB binds at a surface-accessible pocket located around subdomains IIB, consistent with reported non-Sudlow binding regions for cationic peptides. When complexed with SDCS, the preferred binding orientation shifted toward a more peripheral, solvent-exposed region, suggesting steric shielding of PMB’s Dab residues and reduced affinity for HSA, as shown in Figure 7. The binding affinities of PMB with HSA showed a negative value of −3.66 kcal/mol compared to PMB-SDCS-HSA of 1.88 kcal/mol. The negative binding energy indicated a spontaneous and favorable interaction between PMB and HSA. In contrast, the positive binding energy for the PMB-SDCS complex with HSA suggests a weak and non-spontaneous interaction, implying that SDCS effectively shields PMB from binding to HAS. These results may postulate that the PMB molecules were shielded by SDCS, resulting in higher drug availability than in the case of free PMB, which is likely to bind with HSA. Hence, the serum concentration of PMB-SDCS was higher than that observed with free PMB in our rat model.

## 3. Discussion

Polymyxins continue to gain interest with the rise of the antimicrobial resistance pandemic. Addressing the nephrotoxicity issue of polymyxins remains a challenge that limits the use of polymyxins in clinical therapy [29]. The therapeutic window of polymyxins is narrow; for example, clinical PK/PD targets for PMB often aim for average steady-state plasma concentrations of 2–4 µg/mL, which overlaps with concentrations known to cause nephrotoxicity [30]. Dosing optimization becomes more important through detailed findings about polymyxin-induced nephrotoxicity to overcome the narrow therapeutic window of polymyxins by increasing its antimicrobial potency and decreasing its nephrotoxicity [31]. Previous studies showed incorporating PMB into SDCS micelles showed increased viability of normal human primary renal proximal tubule epithelial cell line while providing equivalent antimicrobial activity when tested against carbapenem-resistant *Acinetobacter baumannii* and resistant *Pseudomonas aeruginosa* [23,26].

In this study, the PMB was formulated with SDCS, with a 1:2 ratio selected based on previous work by Madhumanchi et al. and Temboot et al., demonstrating that SDCS micelles significantly reduced PMB-induced cytotoxicity and hemolysis in human renal epithelial and erythrocyte models [23,26]. The PMB-SDCS formulation produced in this research has hydrodynamic properties similar to those in the previous report and produces a clear solution when suspended in water. The formulations were subcutaneously administered to a group of rats on a 7-day treatment course at a dosage of 6 mg/kg/day of PMB equivalent and compared to PMB, SDCS (carrier), and normal saline. In our rat model, after seven days of treatment, the group subjected to PMB treatment experienced a significantly reduced body weight and body weight progression for some individual rats. The observed weight loss in the PMB group likely reflects multiple interacting mechanisms, including renal tubular injury and altered metabolism. The elevated BUN and creatinine levels support impaired renal function, while histopathological evidence of tubular degeneration corroborates tissue-level damage. These findings align with decreased nutrient assimilation and possible anorexic effects secondary to systemic inflammation, explaining the reduced body weight gain in PMB-treated rats. In contrast, PMB-SDCS mitigated these alterations, consistent with normal renal biomarkers and preserved tissue integrity [32].

The nephrotoxicity of polymyxin can be detected or defined by elevated serum Cr and BUN, which are commonly used to measure glomerular damage [33]. The rat groups administered with 6 mg/kg/day of PMB showed elevated BUN and Cr after seven days of administration. This level might not be significantly elevated depending on the degree of injury to renal glomeruli [34]. Polymyxin-induced kidney injury is strongly associated with oxidative stress, which is driven by increased reactive oxygen species (ROS) production after exposure [35,36]. In the PMB group, the serum SOD and CAT levels showed reduced oxidative stress enzyme activity, which was consistent with previous findings [37]. Deficiencies in key antioxidant enzymes, such as SOD and CAT, have been shown to exacerbate kidney damage. SOD deficiency aggravates renal dysfunction, promotes tubulointerstitial fibrosis, induces inflammation, and increases apoptosis in kidney tissues. Similarly, CAT deficiency, as observed in a mouse model of unilateral ureteral obstruction, worsens tubulointerstitial fibrosis and elevates lipid peroxidation products that further contribute to tubulointerstitial injury [38]. PMB formulated with SDCS significantly mitigated nephrotoxicity and did not hinder kidney function as evidenced by comparable BUN and Cr levels. Furthermore, lower ROS production after exposure was indicated by higher activity of SOD and CAT.

The serum biochemistry results were in line with the histopathological changes in rat kidneys after seven days of treatment with 6 mg/kg/day of PMB. The results showed signs of congested blood vessels due to inflammation indicated by inflammatory cells present from the histology of the glomerulus in the renal cortex. The affected glomerulus exhibited signs of hyperemia characterized by dilated capillaries engorged with red blood cells. This finding suggests increased blood flow to the glomerulus that is potentially part of an inflammatory response or hemodynamic alteration where oxidative stress can trigger transcription factors that activate genes driving inflammatory pathways [39,40]. The administration of SDCS showed no histological damage on the rat kidney with alteration that was similar to the control group. The PMB formulation attenuated kidney damage from PMB exposure with mild alternations without evidence of renal glomerular injury. The inflammation caused by increased ROS production was also apparent from the histological alteration of the liver, lung, and spleen tissues. PMB-treated animals showed significant liver damage that included monocyte infiltration, necrotic foci near blood vessels, and disrupted hepatocyte architecture. In contrast, the PMB formulation group exhibited only mild monocyte infiltration with preserved liver structure. The PMB-treated rats showed increased small pulmonary venules with fibrin thrombi that indicated endothelial damage along with a higher number of multinucleated giant cells in the spleen. In contrast, the PMB formulation-treated animals exhibited fewer thrombi and multinucleated giant cells in both the lungs and spleen.

PMB undergoes extensive reabsorption in the renal tubule, and a very high concentration of PMB is found in human renal tubular cells compared to the extracellular concentration. The increased accumulation of PMB leads to cell swelling and lysis by disrupting the permeability of the renal tubular epithelial cell membrane [41,42]. In our results, the administration of the PMB formulation increased the serum concentration profile of PMB. The results showed a higher onset concentration compared to pure PMB. The higher free-PMB from formulation in the serum is in line with a significantly lower concentration of PMB in the kidney tissue after 24 h resulting in more free-PMB available from the loss of PMB renal uptake. The accumulation of PMB in the kidney might be higher depending on the timing of sample collection. In another study, the PMB kidney/serum concentration ratio was 18-fold [7]. The decrease in kidney accumulation in this current study might be attributed to the altered physicochemical properties of the PMB-SDCS complex. The overall negative surface charge of the SDCS micelles may reduce the electrostatic attraction to anionic phospholipids on the brush-border membrane of renal tubular cells, a key mechanism for the high renal reabsorption and accumulation of free, cationic PMB. This is consistent with findings for other negatively charged nanocarriers, such as colistin liposomes, which also demonstrated reduced renal accumulation [43]. The molecular docking results provide a plausible mechanism for the observed increase in serum total PMB concentration. The shielding of PMB by SDCS, leading to a highly unfavorable (positive) binding energy with HSA, suggests that less PMB is bound to serum albumin in the circulation. While our assay measured total PMB, a shift towards the unbound fraction could alter the drug’s distribution and clearance kinetics. The increased unbound fraction, coupled with the reduced affinity for renal tubular cells due to the complex’s negative charge, collectively explains the higher serum levels and lower renal accumulation. Whereas another study suggests that the electrostatic interaction played a major role, mainly from the close contact of the protein with dab residues of polymyxins [44]. Unlike PMB, which preferentially accumulates in renal cortex tissue, PMB nonapeptide distributes more uniformly throughout the kidney [9]. The PMB-SDCS formulation mimics this favorable distribution pattern by minimizing localized cortical accumulation, possibly through electrostatic repulsion and reduced endocytic uptake. The PMB-SDCS interaction showed that the main interaction from dab residues was shielded by SDCS, with different binding sites also contributing to significantly lower binding affinity with the HSA. The shielding effect of the higher availability of free PMB in serum and the reduced kidney accumulation may potentially increase the therapeutic window of PMB.

Although this study focused on safety and biodistribution, previous reports confirmed that SDCS formulations preserve PMB’s antibacterial potency in vitro [23,26]. Future in vivo infection studies are warranted to confirm that the safety improvement does not compromise antimicrobial efficacy. A limitation of this work is that the molecular docking was performed using human serum albumin, whereas the in vivo studies used rats. Structural differences between HSA and rat serum albumin may lead to minor variations in binding affinity; however, the overall interaction trends are expected to be consistent. This study was limited by its short treatment duration and use of a healthy rat model, which may not fully represent infected physiological conditions. In addition, only preliminary pharmacokinetic and histological endpoints were assessed, without direct in vivo antimicrobial efficacy data. Nevertheless, the observed reduction in nephrotoxicity and improved serum exposure suggest a promising foundation for translational development. Future studies should evaluate PMB-SDCS in infection models and explore long-term safety and pharmacodynamic correlations to support potential clinical application.

## 4. Materials and Methods

### 4.1. Materials

Polymyxin B sulfate and deoxycholic acid were purchased from Sigma-Aldrich (St. Louis, MO, USA). SDCS was synthesized in-house (Gangadhar et al., 2014) [21]. Superoxide dismutase and catalase kits were obtained from Cayman Chemical (Ann Arbor, MI, USA). Polyamide membranes with pore sizes of 0.22 µm and 0.45 µm were obtained from Sartorius (Göttingen, Germany). All chemicals, except tetrahydrofuran, were used as received without further purification. All other reagents and chemicals were of analytical grade.

### 4.2. Preparation and Characterization of the PMB-SDCS Formulation

The PMB-SDCS micelles were prepared using 80 mg of PMB (0.06 mmol) and 58 mg of SDCS (0.12 mmol), corresponding to a 1:2 molar ratio (PMB:SDCS) based on their molecular weights (PMB ≈ 1302 g/mol; SDCS ≈ 480 g/mol). This ratio was optimized in previous studies [23,26]. The mixtures were stirred until complete dissolution. To these solutions, 5 mL of a sodium hydroxide solution (0.2 M) was added slowly dropwise at room temperature to obtain a clear solution. The pH of the solution was about 9.5, which was adjusted to 7.4 using phosphoric acid (0.2 M) for an in situ phosphate buffer. The final volume of the solution was made by adding deionized water. The solution was lyophilized, and the reconstituted formulation particle size and zeta potential were measured using a Zetasizer Nano ZS (Malvern, Worcestershire, UK). The drug content was also measured using liquid chromatography-mass spectrometry (LC-MS). The osmolarity of the formulation in normal saline was measured using a K-7400S freezing point osmometer (Knauer GmbH, Berlin, Germany).

### 4.3. Animals

The animal study was approved by the Animal Ethics Committee of Prince of Songkla University (Project License No. MHESI 68014/675, Ref. AR042/2024). The animals were housed in a controlled environment with a temperature of 22 ± 2 °C, relative humidity of 50 ± 10%, and a 12/12-h light/dark cycle. They were provided with a standard laboratory rodent diet and water ad libitum. The study was conducted on male outbred Sprague-Dawley rats (5–7 weeks old, 130–170 g) that were obtained from the National Laboratory Animal Center, Mahidol University, Nakorn Pathom, Thailand. The rats were fed the standard feed protocol by the Southern Laboratory Animal Facility, Faculty of Sciences, Prince of Songkla University. Rats were allowed to acclimate for 7 days prior to the experiment. Animals that did not reach a body weight of 110 g by the end of the acclimatization period were planned to be excluded; however, all rats met the inclusion criteria and were included in the study.

### 4.4. Study Design

The nephrotoxicity experiment of the PMB formulation was performed on 36 male rats that were randomly divided into four groups (*n* = 9). Randomization was performed using computer-generated random sequences in Microsoft Excel with a fixed seed value to ensure reproducibility. Block randomization was used to balance the number of animals per group. Each group was treated subcutaneously for ease of administration and controlled dosing with 6 mg/kg/day of polymyxin B sulfate (Group 1: positive control group), PMB-SDCS formulation (Group 2), SDCS (Group 3: carrier), and normal saline solution (Group 4: normal control) for seven consecutive days. The PMB dose of 6 mg/kg/day (expressed as PMB equivalent) was selected based on previous pharmacokinetic and toxicity studies in rats, where this dose consistently produced measurable nephrotoxicity without lethality [7,34]. All formulations were administered subcutaneously in a constant injection volume of 1 mL/kg using normal saline as the vehicle, ensuring comparable dosing across groups. The average weight gain of each group was recorded for the experiment. We randomized the treatment order and controlled the timing of measurements, and all researchers involved in the study were blinded to the intervention. The animals were euthanized 24 h after the last treatment with an intraperitoneal lethal dose of sodium pentobarbital (200 mg/kg). Blood samples were collected by cardiac puncture, and the serum was separated by centrifugation (3000× *g* for 15 min) and stored at −80 °C until assayed. Samples of the kidney, liver, spleen, lung, and heart tissues were carefully collected, weighed, and sectioned for histopathological analysis. Histological observation was performed to assess potential tissue damage and evaluate the extent of toxicity following the treatment.

### 4.5. Biochemical Analysis

The serum BUN and Cr levels were measured using an autoanalyzer to investigate the effects of the PMB-SDCS formulation and PMB after seven days of consecutive treatments on the physiology of the kidneys and liver. The serum levels of SOD and CAT were measured after the 7-day treatment period to investigate the oxidative properties of the treatments. The measurements were carried out using commercial kits according to the manufacturer’s instructions.

### 4.6. Histopathological Evaluation

Excess fat from the kidney, liver, spleen, lung, and heart tissues was removed through dissection and trimming. All samples were preserved in 10% buffered formalin using a fixative volume at least 10 times the tissue volume for three days. Subsequently, the tissues were dehydrated through a graded series of ethanol (70%, 80%, 95%, and 100%) using a tissue processor (LEICA TP 1020, Leica Microsystems GmbH, Wetzlar, Germany), with each concentration step lasting for 60 min. Following this, the tissues were embedded in Paraplast blocks using a tissue embedder (LEICA EG 1160, Leica Microsystems GmbH, Wetzlar, Germany). Thin sections that measured 5 μm in thickness were obtained using an automatic microtome. The slides were stained with Harris’ hematoxylin and eosin (H&E) and examined under an Olympus DP73 microscope (Olympus, Tokyo, Japan) equipped with cellSens software version 6.1.4.2. Histopathological analysis was performed by an investigator who was blinded to the treatment groups.

### 4.7. Biodistribution and Serum Concentration of PMB

For the biodistribution study, a sample size of four rats per group was chosen as a preliminary exploratory analysis to determine major organ distribution trends. This sample size was consistent with previous biodistribution studies involving polymyxins and sufficient to identify statistically meaningful differences in renal accumulation [7]. Two groups of rats (*n* = 4) were subcutaneously administered either PMB or the PMB-SDCS formulation as a single administration group. At predetermined time points (2, 4, 6, and 24 h post-dose), approximately 150 µL of blood was collected from the lateral tail vein using a sterile 25-gauge needle and capillary tubes under gentle manual restraint. The tail was warmed to dilate the vein, and alternating sides were used for repeated sampling. Blood was collected into serum separator tubes, allowed to clot at room temperature for 30 min, and centrifuged at 3000× *g* for 10 min at 4 °C. The resulting serum was stored at −80 °C until analysis. The rats were euthanized at 24 h to collect the kidney, liver, spleen, lung, and heart samples. PMB concentrations were measured in LC-MS for free (unbound) fraction of serum and tissue homogenates after protein precipitation.

### 4.8. PMB Assay on Serum and Organ Using LC-MS

The PMB was quantified using an LC-MS/ion trap time-of-flight system (Shimadzu, Tokyo, Japan) equipped with a C18 column (2.1 mm id × 150 mm, 2.7 µm particle size; Thermo Fisher Scientific, Scoresby, Australia). Mass spectrometry was employed with mass-to-charge ratio detection conducted from *m*/*z* 200–1200 in positive ionization mode (ESI+). The column temperature was set at 35 °C. The tuning voltage was fixed to 1.68 kV. The interface, curved desolvation line, and heat block temperature were maintained at 200 °C. Nitrogen gas flowed at a rate of 1.5 L/min. The mobile phase was a mixture of 0.1% formic acid and 0.01% trifluoroacetate (TFA) in acetonitrile/water (25:75 *v*/*v*) and pumped at 0.2 mL/min for a run time of 10 min with an injection volume of 10 µL of standard PMB. The organ samples were weighed and homogenized with water (2 times their weight), and 200 µL of organ and serum were added to 400 µL of 0.1% TFA in acetonitrile to precipitate the protein. The mixture was vortexed for 30 s and centrifuged at 7200× *g* for 5 min. A quantity of 400 µL of supernatant was added to 600 µL of the mobile phase. A known amount of standard PMB in blank serum was prepared using the same method to produce a calibration curve of PMB and quantification was performed using colistin sulfate (1 µg/mL) as the internal standard. The monitored mass transitions were *m*/*z* 602.4 → 101.1 for PMB and *m*/*z* 585.5 → 101.1 for colistin. The calibration curve was linear over the range of 0.05–10 µg/mL (R^2^ = 0.998). The recovery of PMB from serum and tissue homogenates was greater than 85%, and the matrix effect was found to be negligible under the used chromatographic conditions.

### 4.9. PMB Binding on HSA by Molecular Docking

PMB and PMB-SDCS binding with HSA was simulated using AutoDock 4.2 from the Scripps Research Institute (http://autodock.scripps.edu/, accessed on 9 September 2025) [18]. The PMB structure was obtained from Chemspider database while the SDCS drawn in Avogadro and joined with PMB to simulate the shielding effect of SDCS. The ligand then docked to HSA obtained from rscb.org (PDB ID: 5TGZ). The pdb file obtained was converted to pdbqt (a molecule with partial charges and atom type). The docking grid box was centered at coordinates (x = 29.551, y = 31.855, z = 23.559) with dimensions 250 × 250 × 250 Å^3^ to cover the entire HSA molecule position to perform blind docking with no specific binding site target. The Lamarckian Genetic Algorithm (LGA) was used with 50 independent runs, a population size of 200, and each docking run involved 200 energy evaluations. The docking was then processed in AutoDockTools using the pdbqt file of the receptor and ligand grid parameter file, and the docking parameter file to produce the docking log file (.dlg). The resultant files were then analyzed and the lowest docking score conformations were analyzed and visualized in Discovery Studio Visualizer Software Version 25.1.0.24284 (BIOVIA, Dassault Systèmes^®^, San Diego, CA, USA). The docking protocol was validated by re-docking the native co-crystallized ligand, which yielded a root-mean-square deviation (RMSD) of <2.0 Å, confirming the reliability of the docking parameters.

### 4.10. Statistical Analysis

The sample size was calculated using the MINITAB Statistical Analysis Package (Minitab 18, Minitab Inc., State College, PA, USA). The sample size was determined using a one-way ANOVA with α = 0.05, an assumed standard deviation of 1.5, and four levels of the factor. With a target power of 0.8, the calculated sample size was 9, achieving an actual power of 0.806. The results were analyzed and expressed as mean ± SD. The data were evaluated using one-way or repeated measures ANOVA, as appropriate, followed by Tukey’s post hoc test for multiple comparisons. The level of significance was set at *p* < 0.05. All statistical comparisons were determined using GraphPad Prism 8 (GraphPad Software, Inc., San Diego, CA, USA).

## 5. Conclusions

PMB was formulated with SDCS, which showed an increased safety profile of PMB in vivo. The produced formulation was administered in a rat model and demonstrated better histopathological changes in the rat kidney, liver, lung, and spleen compared to standard PMB. The reduced nephrotoxicity was indicated by the measurement of serum biochemistry markers, which showed improved kidney function and lowered oxidative stress. The PMB formulation increased the PMB serum concentration which was supported by the HSA binding simulation while decreasing accumulation in the kidney, suggesting a potentially improved therapeutic window that warrants further investigation. Future studies using infection models can be conducted as a step toward advancing to clinical trials.

## Figures and Tables

**Table 1:** Physical properties of the PMB-SDCS formulation (*n* = 3) (mean ± SD).

Formula	Particle Size (nm)	Zeta Potential (mV)	Drug Content (% *w*/*w*)	Osmolarity ^1^ (mOsm/Kg)
PMB-SDCS ratio 1:2	193 ± 27	−5.84 ± 1.32	57.27	311 ± 2

^1^ osmolarity reconstituted in normal saline.

## Data Availability

The original contributions presented in this study are included in the article. Further inquiries can be directed to the corresponding author.

## Abbreviations

The following abbreviations are used in this manuscript:

PMB	Polymyxin B
SDCS	Sodium Deoxycholate sulfate
HSA	Human Serum Albumin
LPS	Lipopolysaccharides
BUN	Blood Urea Nitrogen
Cr	Creatinine
SOD	Superoxide Dismutase
CAT	Catalase
